# Undiagnosed Perihepatic Abscess Revealed at Autopsy of a Patient With COVID-19 Undergoing Prolonged Extracorporeal Membrane Oxygenation (ECMO) Therapy

**DOI:** 10.7759/cureus.58338

**Published:** 2024-04-15

**Authors:** Issei Seike, Kengo Oshima, Hiroaki Baba, Hajime Kanamori, Tetsuji Aoyagi

**Affiliations:** 1 Department of Infectious Diseases, Internal Medicine, Tohoku University Graduate School of Medicine, Sendai, JPN; 2 Division of Infection Control, Tohoku University Hospital, Sendai, JPN; 3 Department of Clinical Microbiology and Infection, Tohoku University Graduate School of Medicine, Sendai, JPN

**Keywords:** cholecystitis, perihepatic abscess, extracorporeal membrane oxygenation, autopsy, covid-19

## Abstract

The use of extracorporeal membrane oxygenation (ECMO) increased during the COVID-19 pandemic. However, complications associated with the long-term use of ECMO are poorly understood.

This case report describes the autopsy findings of a perihepatic abscess in a patient with long-term COVID-19, which could not be diagnosed before death. In cases where the source of infection remains elusive but uncontrolled infections occur, we recommend the combined use of ultrasonography and contrast-enhanced computed tomography (CT) in patients with COVID-19 undergoing prolonged ECMO support, with particular consideration given to the potential development of cholecystitis.

## Introduction

Extracorporeal membrane oxygenation (ECMO) is used to manage patients severely ill with COVID-19. The COVID-19 pandemic has led to a significant increase in the use of ECMO [1]. Moreover, the duration of ECMO support for patients with COVID-19 has been extended from 14.1 to 20.0 days [2], and cases of recovery even after prolonged ECMO use have been documented [3].

Autopsies of patients with COVID-19 undergoing ECMO revealed an increase in bleeding events, including intracranial hemorrhage [4]. However, studies on infectious diseases discovered during autopsies of patients with COVID-19 undergoing ECMO have been limited. Although disseminated mucormycosis and cholangitis from intestinal ischemia have been detected during autopsies [5,6], occurrences of cholecystitis or perihepatic abscesses have not been documented. In this report, we present a case in which a perihepatic abscess was discovered during the autopsy of a patient severely ill with COVID-19 who had undergone prolonged ECMO therapy. Remarkably, the abscess remained undetected on non-contrast computed tomography (CT) imaging.

## Case presentation

A 69-year-old male was admitted to the hospital with dyspnea. The patient had a medical history of diabetes, hypertension, myocardial infarction, and colon cancer. Additionally, the patient had a significant smoking history, with an average of 80 cigarettes per day for 22 years. He had received two doses of the SARS-CoV-2 vaccine, the last being seven months prior to hospital admission. On admission, the patient entered respiratory failure, required intubation, and was subsequently transferred to Tohoku University Hospital, Japan. The PCR test confirmed the presence of the Omicron Ba.1 variant.

**Figure 1:**
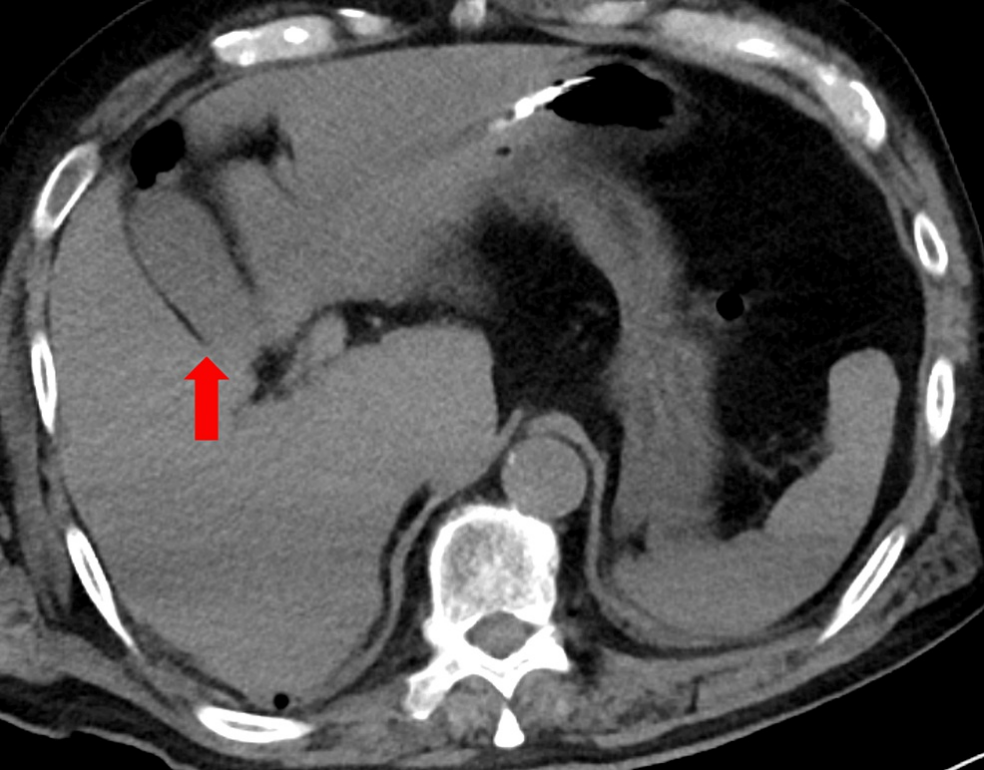
Computed tomography of the gallbladder upon admission There is no gallbladder swelling or bile mud (arrow).

Upon admission, the patient’s vital signs were as follows: blood pressure, 117/94 mmHg; pulse rate, 104 bpm; and oxygen saturation (SpO_2_), 80% during tracheal intubation. Reticular skin lesions were observed throughout the body. The patient’s complete blood count indicated a white blood cell count (WBC) of 20,000/µL with 75.0% neutrophils, a hemoglobin level of 12.3 g/dL, and a platelet count of 7.8×10^4^/µL. Serum biochemistry revealed a C-reactive protein level of 24.2 mg/dL, accompanied by hepatic and renal dysfunction and coagulation abnormalities. CT revealed extensive ground-glass opacities and consolidation in both lungs. No apparent abnormalities were found in the gallbladder or the surrounding tissues (Figure 1).

After admission, the patient's systolic blood pressure dropped to the seventies, necessitating vasopressors, including noradrenaline, adrenaline, dobutamine, and vasopressin, followed by the introduction of ECMO. The patient's respiratory condition initially improved, leading to the discontinuation of ECMO on the seventh day of hospitalization. However, on the eleventh day, the patient experienced fever recurrence and a deteriorating respiratory condition, prompting the reinitiation of ECMO support.

**Figure 2:**
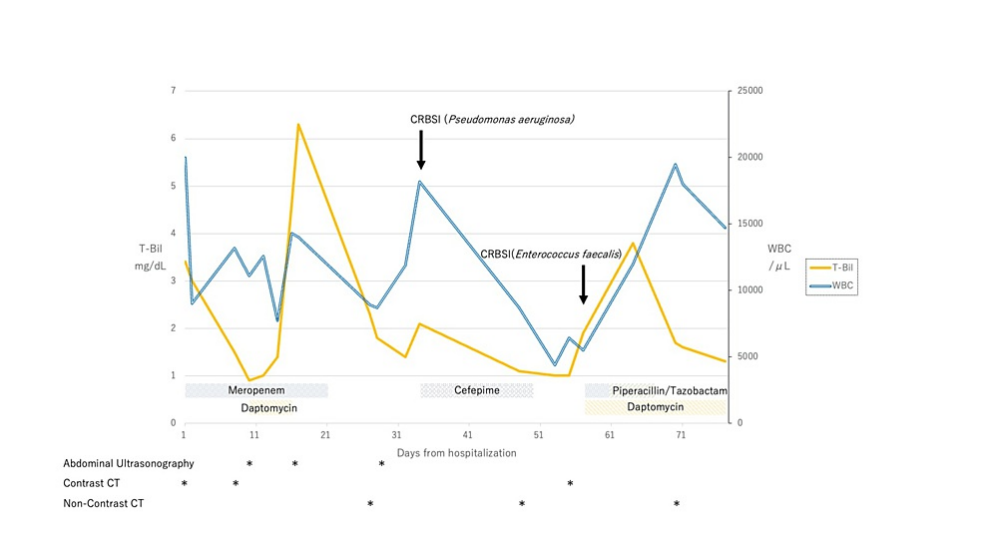
T-Bil, WBC count, timing of imaging studies, and details of antimicrobial usage and durations during hospitalization The patient was diagnosed with CRBSI caused by *Pseudomonas aeruginosa* and *Enterococcus faecalis*. Prior to the patient’s death, difficulties in controlling blood pressure were encountered, despite a decreasing trend in WBC count. *Escherichia coli* and *Streptococcus anginosus* detected in the perihepatic abscess were not covered by the antimicrobial agents. T-Bil: total bilirubin, WBC: white blood cell, CRBSI: catheter-associated bloodstream infection

**Figure 3:**
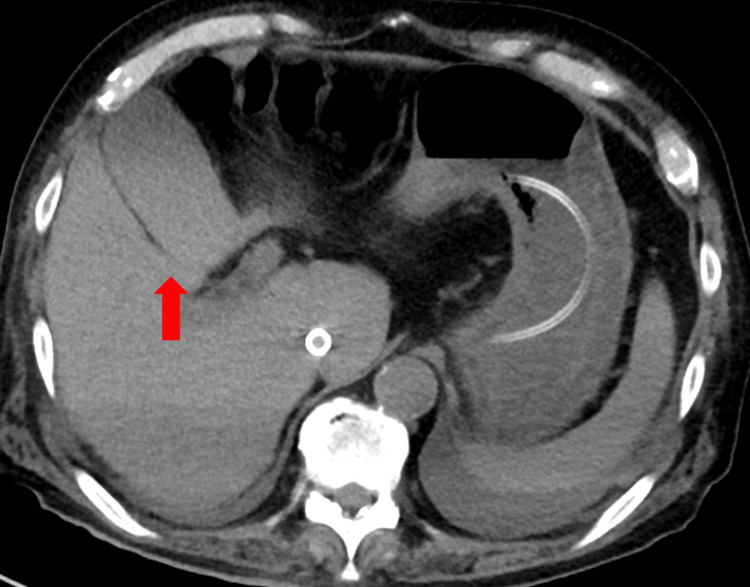
Computed tomography of the gallbladder on the twenty-seventh day Enlargement of the gallbladder is evident, with a pale hyperabsorptive zone indicating potential bile concentration or the presence of bile sludge (arrow).

On the tenth day of hospitalization, the patient exhibited fever and worsening respiratory symptoms, leading to the initiation of meropenem and daptomycin (Figure 2). Daptomycin was chosen over vancomycin due to worsening renal function. Subsequently, total bilirubin levels increased, although abdominal ultrasonography did not reveal any signs of cholecystitis. On the twenty-seventh day, as the inflammation subsided, CT showed gallbladder enlargement, indicative of bile stasis and sludge (Figure 3).

Throughout hospitalization, the patient experienced recurrent bleeding and infectious episodes, including heparin-induced thrombocytopenia, gastrointestinal bleeding, and alveolar hemorrhage. Tube feeding was stopped during episodes of gastrointestinal bleeding. The patient developed a catheter-related bloodstream infection caused by *Pseudomonas aeruginosa*.

**Figure 4:**
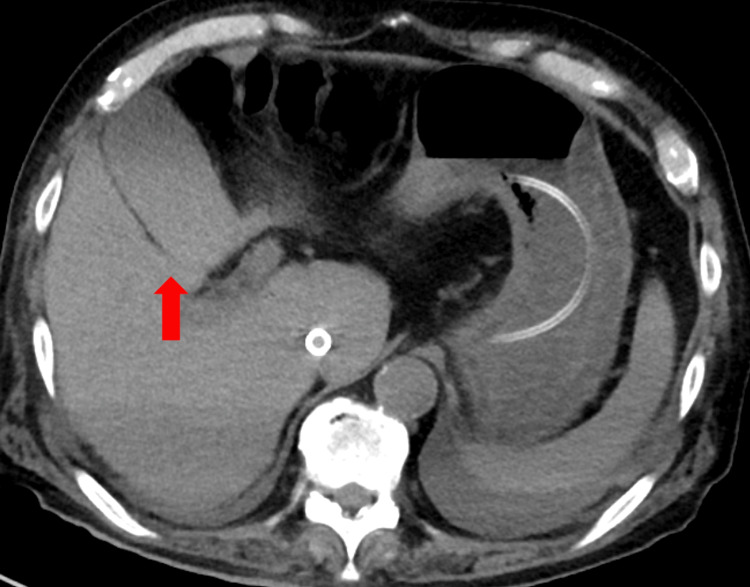
Computed tomography of the gallbladder on day 70 The gallbladder is enlarged; however, no perihepatic abscess is observed (arrow).

On day 57, fentanyl was switched to morphine to reduce respiratory effort for weaning from ECMO. However, on the same day, the patient developed septic shock accompanied by elevated total bilirubin levels. Meropenem and daptomycin were then administered. Catheter-associated bloodstream infection was diagnosed based on the detection of *Enterococcus faecalis* on the tip of a peripherally inserted central venous catheter and in two sets of blood cultures. On day 68, the antibacterial regimen was modified to daptomycin monotherapy. As the treatment of catheter bloodstream-associated infections progressed, the patient’s respiratory status improved, and weaning from ECMO was considered. A non-contrast CT scan conducted on day 70 did not reveal any evident abnormal parenchymal brain changes. Although the gallbladder remained enlarged, no significant alterations were noted (Figure 4). However, on day 74, the patient’s blood pressure decreased, and blood pressure control became difficult. The patient died on day 80 of hospitalization.

**Figure 5:**
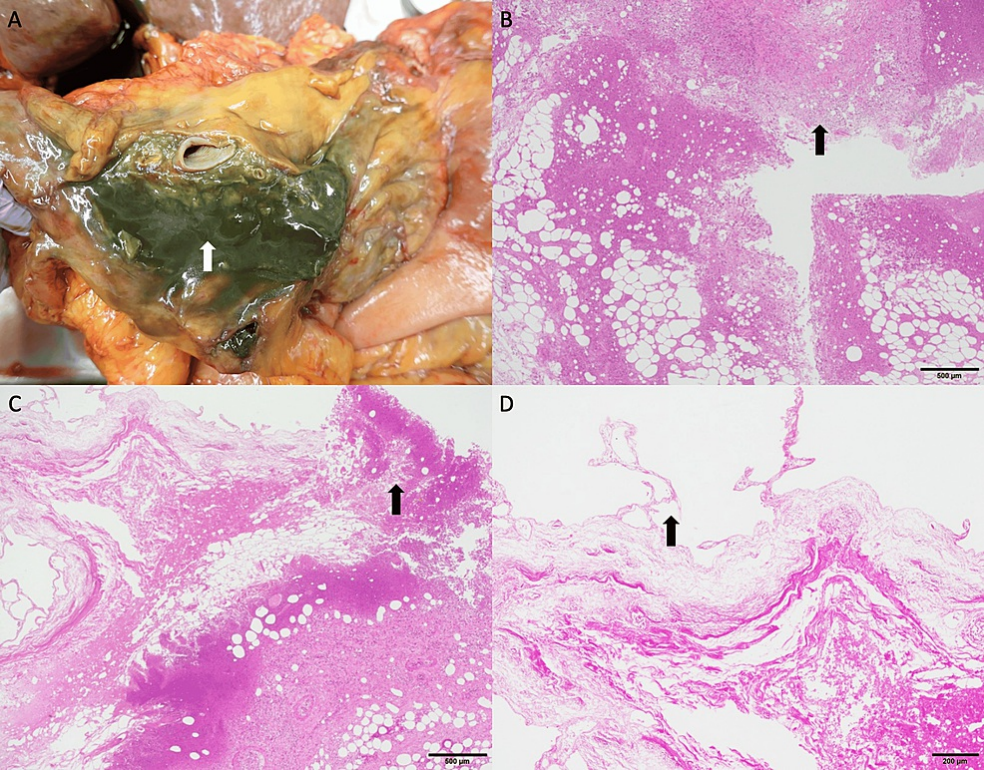
Autopsy findings (A) Perihepatic abscess (arrow) detected during autopsy. (B) H&E stain. Histopathological examination of the perihepatic abscess (arrow; scale bar 500μm). (C) H&E stain. The abscess extends continuously from the gallbladder's mucosa (arrow; scale bar 500 μm). (D) H&E stain. Ischemic alterations (arrow) are noted in the gallbladder mucosa (scale bar 200 μm). H&E: Hematoxylin-eosin

Due to difficulties in controlling blood pressure with vasopressors, an autopsy was conducted to investigate whether an undiagnosed infection was the underlying cause of the issue. During autopsy, the gallbladder appeared enlarged, with an abscess noted in proximity to the lesser omentum and gallbladder (Figures 5A, 5B). Adhesions were observed between the hilar region and ascending colon, with the serosal surface displaying a bile-colored appearance. The abscess was continuous with the mucosal layer of the gallbladder (Figure 5C). The gallbladder showed necrosis of the mucosa, which was indicative of acalculous cholecystitis (Figure 5D). A bacterial culture of purulent material revealed the presence of *Escherichia coli* and *Streptococcus anginosus* with favorable susceptibility.

## Discussion

In this case, the autopsy revealed a perihepatic abscess in a severely ill patient with COVID-19 who had undergone prolonged ECMO therapy. During treatment, ultrasonography, non-contrast CT, and contrast CT were performed; however, cholecystitis was not diagnosed. An abscess resulting from cholecystitis remained undetected on non-contrast CT before the patient’s death.

Pathological examination revealed that the perihepatic abscess had originated in the gallbladder and displayed signs of ischemia. Because of the preserved structure of the gallbladder wall, necrosis due to ischemia, rather than necrosis due to infection, was considered. Therefore, the patient developed acalculous cholecystitis due to ischemia of the gallbladder, which eventually led to necrosis of the gallbladder wall and the formation of a perihepatic abscess. This change to daptomycin monotherapy may have resulted in inadequate coverage of the causative organism of cholecystitis, leading to sepsis and death.

The incidence of gallbladder disease in the intensive care unit has been reported to increase from 1.6% without ECMO to 9.2% with ECMO management [7]. Ischemia, hemolysis, parenteral nutrition, opioid therapy, vasoactive drugs, and prolonged ECMO therapy are known risk factors [8,9]. A higher incidence of 7.4% has been reported in patients treated with ECMO. Hemolysis and ischemia due to ECMO are considered causes, and long-term treatment is a risk factor [7,10]. Acalculous cholecystitis has been reported in 7% of patients with COVID-19 receiving ECMO for >50 days [3]. Cholecystitis may be underdiagnosed because of a lack of autopsies, as seen in this case. Because the risk of cholecystitis is increased in patients undergoing long-term ECMO [7], it is considered to occur more frequently. In this particular case, gastrointestinal bleeding made it difficult to control the thrombus and interrupted tube feeding. This could be a complication of ECMO [11], further increasing the risk of acalculous cholecystitis. Although no evident thrombus was found in the gallbladder tissue, repeated ECMO circuit exchanges due to thrombus formation suggested gallbladder necrosis from the microthrombi. Acalculous cholecystitis can lead to gangrenous cholecystitis [12], potentially leading to abscess formation.

A non-contrast CT scan was performed on day 70; however, acalculous cholecystitis and a perihepatic abscess remained undiagnosed until autopsy. According to the 2018 Tokyo Guidelines, a diagnosis of acute cholangitis requires local and systemic signs of inflammation and imaging findings [13]. In patients with COVID-19 receiving ECMO support, the maintenance of constant blood temperature can mask the onset of fever. Physical examination is also challenging owing to the lack of communication because of sedation. Additionally, ECMO limits patient mobility for inspection.

In this case, acalculous cholecystitis and a perihepatic abscess were not detected until the autopsy. The diagnosis of cholecystitis in patients treated with ECMO is challenging [7]. Ultrasonography is reportedly inferior to abdominal CT in terms of sensitivity [14]. Although ultrasonography failed to identify the gallbladder in this case, the procedure was easy to perform and could be repeated. While contrast-enhanced CT is effective in confirming cholecystitis and diagnosing abscesses, its utility in patients on ECMO is hampered by mobility constraints [15,16]. Using both ultrasonography and contrast-enhanced CT is crucial to enhance diagnostic sensitivity. Although open surgery is challenging during ECMO, percutaneous transhepatic gallbladder drainage has been performed [7]. With a diagnosis of cholangitis or an abscess, the patient can be switched to a more appropriate antimicrobial agent.

In this study, the autopsy enabled us to identify acalculous cholecystitis and perihepatic abscess as complications in COVID-19 patients on ECMO for an extended period. However, this is based on a single case, emphasizing the importance of accumulating more autopsy cases in the future.

## Conclusions

It is extremely difficult to identify acalculous cholecystitis and perihepatic abscesses for the reasons discussed above, specifically in COVID-19 patients on ECMO. However, a failure to diagnose these conditions accurately could result in the patient's death. Therefore, it is important to perform both ultrasonography and contrast-enhanced CT to detect hidden cholecystitis or perihepatic abscesses when the clinical course differs from the expected, such as the development of hypotension that cannot be controlled with catecholamines.
